# 5-Demethylnobiletin mediates cell cycle arrest and apoptosis *via* the ERK1/2/AKT/STAT3 signaling pathways in glioblastoma cells

**DOI:** 10.3389/fonc.2023.1143664

**Published:** 2023-04-17

**Authors:** Xuehua Zhang, Leilei Zhao, Jinlong Xiao, Yudi Wang, Yunmeng Li, Chaoqun Zhu, He Zhang, Yurui Zhang, Xiao Zhu, Yucui Dong

**Affiliations:** ^1^ Department of Immunology, Binzhou Medical University, Yantai, China; ^2^ School of Computer and Control Engineering, Yantai University, Yantai, China; ^3^ Department of Immunology, Qiqihar Medical University, Qiqihar, China

**Keywords:** glioblastoma, 5-Demethylnobiletin, cell cycle arrest, cell apoptosis, ERK1/2, AKT, STAT3

## Abstract

5-Demethylnobiletin is the active ingredient in citrus polymethoxyflavones that could inhibit the proliferation of several tumor cells. However, the anti-tumor effect of 5-Demethylnobiletin on glioblastoma and the underlying molecular mechanisms are remains unknown. In our study, 5-Demethylnobiletin markedly inhibited the viability, migration and invasion of glioblastoma U87-MG, A172 and U251 cells. Further research revealed that 5-Demethylnobiletin induces cell cycle arrest at the G0/G1 phase in glioblastoma cells by downregulating Cyclin D1 and CDK6 expression levels. Furthermore, 5-Demethylnobiletin significantly induced glioblastoma cells apoptosis by upregulating the protein levels of Bax and downregulating the protein level of Bcl-2, subsequently increasing the expression of cleaved caspase-3 and cleaved caspase-9. Mechanically, 5-Demethylnobiletin trigged G0/G1 phase arrest and apoptosis by inhibiting the ERK1/2, AKT and STAT3 signaling pathway. Furthermore, 5-Demethylnobiletin inhibition of U87-MG cell growth was reproducible *in vivo* model. Therefore, 5-Demethylnobiletin is a promising bioactive agent that might be used as glioblastoma treatment drug.

## 1 Introduction

Glioblastoma (GBM) is the most common, lethal, and aggressive adult central nervous system (CNS) primary malignant brain tumor (1). The traditional treatment of standard for patients with GBM consists of maximum surgical excision followed by concurrent chemotherapy and radiotherapy (2). Unfortunately, the traditional treatment methods are not ideal, the recurrence rate is high as well as the emergence of tumor resistance. After treatment, the median survival time for GBM patients is 15 months, with a 5-year survival rate of less than 5% (3). Therefore, it is essential to discover novel drugs and identify new therapeutic strategies to enhance GBM therapy.

Polymethoxyflavones (PMFs) are a class of highly methoxylated flavonoids unique to citrus plants, and they are gaining increasing attention due to their diverse biological activities (4–6). Nobiletin and tangeretin are the main PMFs in citrus peels (7). Previous research has shown that nobiletin has potent anti-leukemic characteristics and has the potential for chemoprevention (8, 9). 5-Demethylnobiletin (5-DMN) is one of the promising nobiletin derivatives, the most plentiful demethylated PMF, which is mainly generated during long-term storage by autohydrolysis of nobiletin in citrus peel. 5-DMN has plenty of biological properties, including anticancer, anti-inflammatory, antioxidant and neuroprotective (7, 10). Interestingly enough, 5-DMN inhibited the growth of various tumor cells more effectively than nobiletin (11). Recent research has demonstrated that 5-DMN has anticancer effects in human leukemia (12), colon (13) and lung cancer (14). Chen et al. (6) discovered that 5-DMN could increase the synthesis of polymerized tubulin and cause cell cycle arrest in the G2/M phase by activating JNK signal in lung cancer cells. In acute myeloid leukemia, 5-DMN inhibited cell proliferation and induced apoptosis by affecting the NF-κB signaling pathway (15). Furthermore, 5-DMN treatment of colon cancer cell line HCT116 cells increased the apoptosis rate of the cells by activating the caspase cascade reaction (16). However, there was no evidence that 5-DMN inhibited GBM cell growth specifically by modulating cell cycle and apoptosis-related signaling pathways. In addition, studies have shown that 5-DMN not only promoted neurocytogenesis and neurogenesis, but also negatively regulated acetylcholinesterase (AChE) activity (17). This means that 5-DMN could through the blood-brain barrier and could be used in the treatment of neurodegenerative diseases and cholinergic abnormalities (18, 19), and may have a pharmacological advantage in the treatment of intracranial tumors.

In our study, the antitumor effects and potential mechanisms underlying the impact of 5-DMN on GBM were investigated for the first time both *in vitro* and *in vivo*. We elucidated that 5-DMN promoted G0/G1 phase arrest and apoptosis in glioblastoma cells by restraining the ERK1/2, AKT and STAT3 signaling pathways. The results of our study will help to evaluate the potential applications of 5-DMN as a clinical agent for glioblastoma.

## 2 Materials and methods

### 2.1 Ethical statement

All animal experiments protocols were designed in strict accordance with the guidelines and approved by the Animal Experiment Ethics Committee of Binzhou Medical University.

### 2.2 Cell culture and reagents

Human glioblastoma cell lines, including U87-MG, A172 and U251 were obtained from the American Type Culture Collection (ATCC) (Manassas, VA, USA). Cells were grown in Dulbecco’s Modified Eagle’s Medium (DMEM, Gibco) with 10% fetal bovine serum (FBS; Vistech) and 1% penicillin/streptomycin (Beyotime, Shanghai, China) at 37°C incubator with 5% CO_2_. 5-Demethylnobiletin was obtained from Selleck (Shanghai, China) and stored in dimethyl sulfoxide (DMSO) at the appropriate concentration of 10 mM. The inhibitors were purchased from Sigma-Aldrich (StLouis, MO, USA), which were kept at optimal concentrations in DMSO.

### 2.3 MTT assays

When cells reached the logarithmic growth phase, they were digested with 0.25% trypsin and adjusted to a density of 6 × 10^4^ cells/ml and 100 μL cell suspension was added to a 96-well plate. The GBM cells were treated for 48 h with 5-DMN at various doses (0, 6.25, 12.5, 25, 50, 100 μM) after cell adhesion. Each well received 20 μL of MTT reagent (0.5 mg/mL, Sigma-Aldrich) and the cells were incubated for 4 h. The supernatant was discarded, and 150 µL of DMSO was added. After 3 times of shaking plate, the absorbance at 490 nm was measured using a microplate reader (DMI3000, Leica, Germany) to determine the cell viability rate.

### 2.4 Live/dead co-staining by Calcein-AM and PI

Different doses of 5-DMN (0, 12.5, 25, 50 μM) or inhibitors were applied after cells attained 80% confluence in a 6-well plate. After 48 h treatment, the Calcein AM/Propidium Iodide (PI) staining kit (Solarbio, Beijing, China) was used to stain the cells. Incubation for 0.5 h at 37°C, PBS was used to wash the cells and representative photograph were captured using Confocal laser scanning microscopy (Carl Zeiss AG, Jena, Germany).

### 2.5 Migration assays

Wound healing experiments were used to measure cell migratory ability. When GBM cells reached the appropriate density, and confluent cells were wounded with a 10-μL pipette tip before being incubated with 5-DMN for 48 h. Six visual fields were randomly selected to observe and quantify the wound closure rate at different time points under the microscope.

### 2.6 Invasion assays

Transwell chambers (Corning, New York, USA) were precoated with Matrigel (BD Biosciences) in a 24-well plate. The serum-starved cells were diluted with serum-free DMEM medium to a density of 1 × 10^6^ cells/ml and 100 μL cell suspension was added to the upper chamber. The lower well contains 600 μL DMEM supplemented with 5% FBS. When GBM cells had adhered to the well, the lower chamber medium was changed to drug-medium containing 5-DMN of 25 or 50 μM and then incubation for 48 h. The GBM cells on the lower surface were fixed and stained with 1% crystal violet (Sigma-Aldrich). Cell invasion rates were quantified in six randomly selected fields and images of the cells were captured at 20 × magnification.

### 2.7 Cell cycle analysis

GBM cells were incubated with four concentrations of 5-DMN for 48 h before being collected, fixed with 70% pre-cooled ethanol and incubated additional 12 h at 4 °C. Cell precipitation were rinsed twice with PBS, RNase and PI were added for DNA staining, and the fluorescence intensity was measured by Flow cytometry (Beckman Coulter, Inc., Brea, CA, USA). The Modfit software (Scribpps Research, La Jolla, CA, USA) was used to analyze the percentage of cells in each phase of the cell cycle.

### 2.8 Cell apoptosis assays

The flow cytometer was used to analyze cell apoptosis using the Annexin V-FITC Apoptosis Assay Kit (Beyotime). GBM cells at the logarithmic growth stage were seeded in 6-well plate before being treated with 5-DMN (0, 12.5, 25, 50 µM) for 48 h. After the cells were collected, loading buffer in moderation was added for suspended cells, followed by 5 μL Annexin V-FITC and 5 μL PI incubation and prepared within 1 h.

### 2.9 Western blotting

Cell protein was harvested using cell lysis buffer (Solarbio) and protein concentration was measured using the BSA protein kit (Solarbio). Protein samples of 30 μg were taken from each lane for 10% sodium dodecyl sulfate-polyacrylamide gel electrophoresis (SDS-PAGE). The proteins were transferred to PVDF membranes, which were incubated with primary antibody at 4 °C overnight. All antibodies were purchased from Cell Signaling Technology (CST, Danvers, MA, USA): anti-Cyclin D1 (1:1000, cat no. 55506), anti-CDK6 (1:1000, cat no. 13331), anti-Bcl-2 (1:1000, cat no. 15071), anti-Bax (1:1000, cat no. 89477), anti-caspase-3 (1:1000, cat no. 9662), anti-cleaved caspase-3 (1:1000, cat no. 9661), anti-caspase-9 (1:1000, cat no. 9508), anti-cleaved caspase-9 (1:1000, cat no. 9509), p-ERK1/2 (1:2000, cat no. 4370), ERK (1:1000, cat no. 4695), p-AKT (1:2000, cat no. 4060), AKT (1:1000, cat no. 4685), p-STAT3 (1: 2000, cat no. 9145), STAT3 (1:1000, cat no. 9139) and β-actin (1:1000, cat no. 4970). After being three times washed in TBST, PVDF membrane was incubated with HRP-conjugated second antibody at room temperature for 2 h. Enhanced chemiluminescence (ECL, Thermo Fisher Scientific, Shanghai, China) was used to visualize the immunocomplexes, and gray-scale values for each band were determined using Image J software.

### 2.10 *In vivo* antitumor activity study

BALB/c female nude mice aged 4-week-old were purchased from SiPeiFu (SPF Biotechnology Co., Ltd, Beijing) and lived in a germfree environment with constant temperature and humidity, suitable for food and water. All mice were slowly injected subcutaneously with U87-MG cells (5 × 10^6^ cells/100 µL) under the armpit of the right forelimb. When the tumors reached 3-4mm in diameter, these nude mice were divided into 5-DMN and control groups, and administered an intraperitoneal (i. p.) injection of 5% DMSO + 40% PEG300 (Selleck) + 5% Tween 80 (Selleck) + 50% water (control) or 5-DMN 3 mg/kg that was dissolved in DMSO, PEG300, Tween 80 and water (5:40:5:50 v/v). Mice were given treatment every other day using 100 μL total volumes. Tumor volumes were assessed every two days and were calculated as 0.5 × length × width^2^. The mice were sacrificed under adequate anesthesia 21 days after treatment.

### 2.11 Statistical analyses

The experiments data was expressed as the mean ± standard deviation (mean ± SD). All statistical analyses were performed with the SPSS version 23.0 software, and graphics were created with the GraphPad Prism 8 program (GraphPad Software, San Diego, CA). The data from two groups were compared using a two-tailed Student’s t-test or a two-way ANOVA. *p < 0.05; **p < 0.01; ***p < 0.001 were considered statistically significant differences.

## 3 Results

### 3.1 5-DMN inhibits the viability, migration and invasion ability of GBM cells *in vitro*

**Figure 1:**
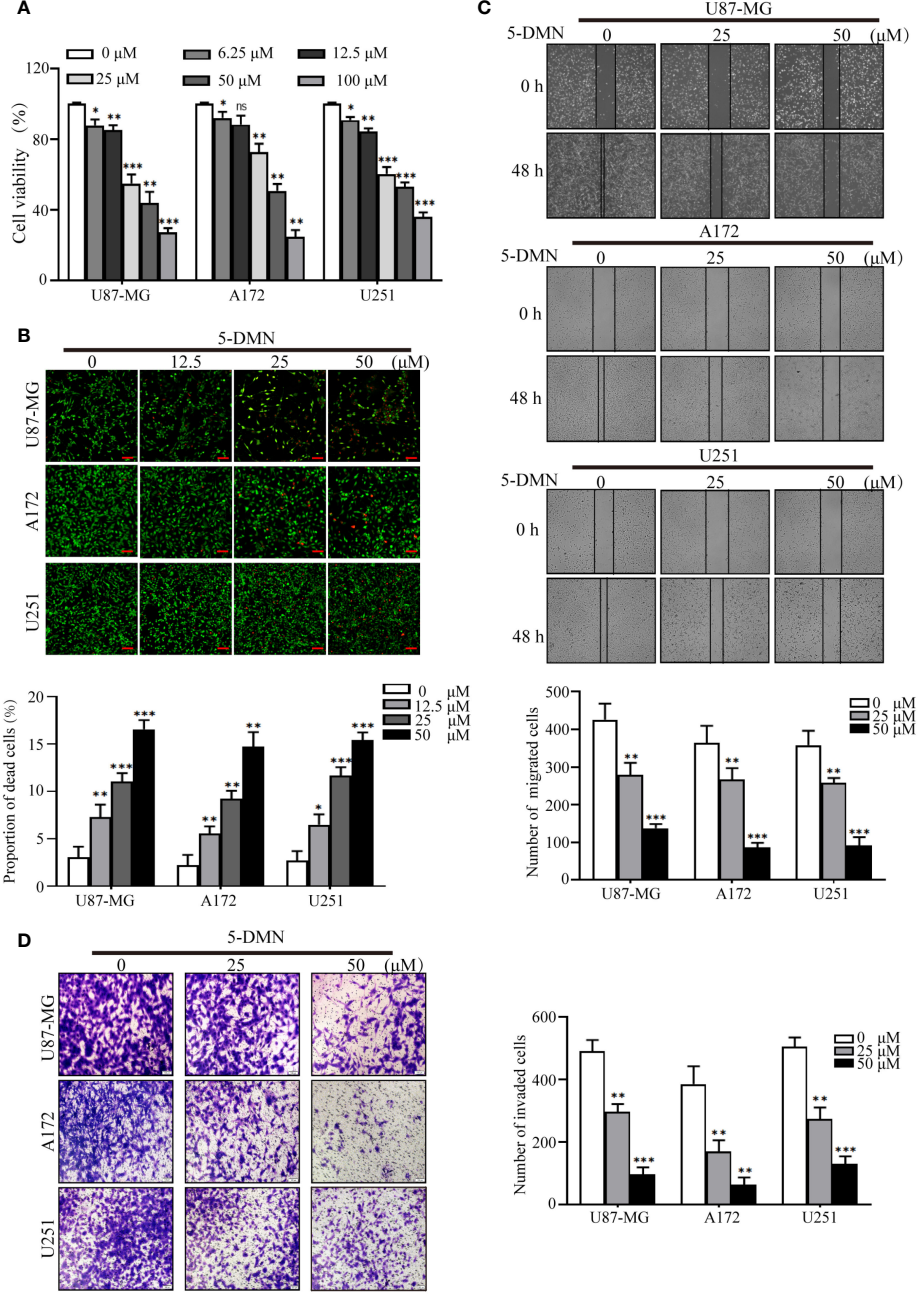
Inhibitory effects of 5-DMN on the viability, migration and invasion of GBM cells. **(A)** Cell viability was determined using the MTT assay after cultured U87-MG, A172, and U251 cells were incubated with various doses of 5-DMN for 48 h. **(B)** The merged image of live cells (green) and dead cells (red) in GBM cells treated with different concentrations of 5-DMN. Scale bar = 100 μm. **(C)** Wound healing assays were used to determine the migration ability of U87-MG, A172, and U251 cells after 48 h of incubation with 5-DMN (0, 25, 50 μM). **(D)** The invasion ability of GBM cells treated with or without 5-DMN (25, 50 μM) for 48 h was detected by transwell assays. Values are the means ± SD of three independent experiments. *p<0.05, **p<0.01, ***p<0.001 vs. cells in the untreated control group. ns, no significance.

To explore the impact of 5-DMN on glioblastoma *in vitro*, U87-MG, A172 and U251 cells were treated with 5-DMN for 48 h at set concentrations. The cell activity data revealed that 5-DMN administration greatly inhibited the proliferation of U87-MG, A172, and U251 cells (**Figure 1A**). Similar procedures were implemented to assess the cell activity on normal human brain astrocyte cell lines (HA1800) and human embryonic kidney (HEK293T) cell lines. The results showed that 5-DMN had no significant cytotoxic activity on HA1800 and HEK293T cell in the concentration range of 0 - 100 μM for 48 h (**Figure S1A**). To determine cell viability more intuitively, a living/dead assay was conducted. Calcein-AM/PI was employed to stain living with green and dead cells with red. The results indicated that the green fluorescence of living cells reduced dramatically, while the red fluorescence increased significantly accompanied the increase of 5-DMN concentration, which further verified the outstanding cytotoxicity of 5-DMN (**Figure 1B**).

The poor prognosis of GBM patients was related to the motility and metastasis ability of the tumor cells. Therefore, migration and invasion experiments were conducted after different concentration 5-DMN (25 μM, 50 μM) was added to U87-MG, A172 and U251 cells. As shown in **Figures 1C, D**, GBM cells migration and invasion in the 5-DMN treatment group was considerably reduced in a dose-dependent manner. In addition, to further evaluate the cytotoxicity on GBM cells of 5-DMN compared with Temozolomide (TMZ), which is the first-line chemotherapeutic drug for patients with GBM, U87-MG, A172 and U251 cells were treated with TMZ for 48 h. As shown in **Figure S1B**, in treatments with the same drug concentration, the cytotoxic effect of TMZ was not obvious in the three GBM cells compared with 5-DMN, suggesting a higher anti-cancer efficacy of 5-DMN in GBM cells. Furthermore, wound healing experiment was conducted after different concentration TMZ (25 μM, 50 μM) was added to GBM cells for 48 h, the results showed that U87-MG, A172 and U251 cells migration was not significantly reduced in the TMZ treatment group (**Figure S1C**). In conclusion, our results demonstrated that 5-DMN had the potential to inhibit GBM cells malignant biological behavior *in vitro*.

### 3.2 5-DMN induces G0/G1 phase cycle arrest in GBM cells

**Figure 2:**
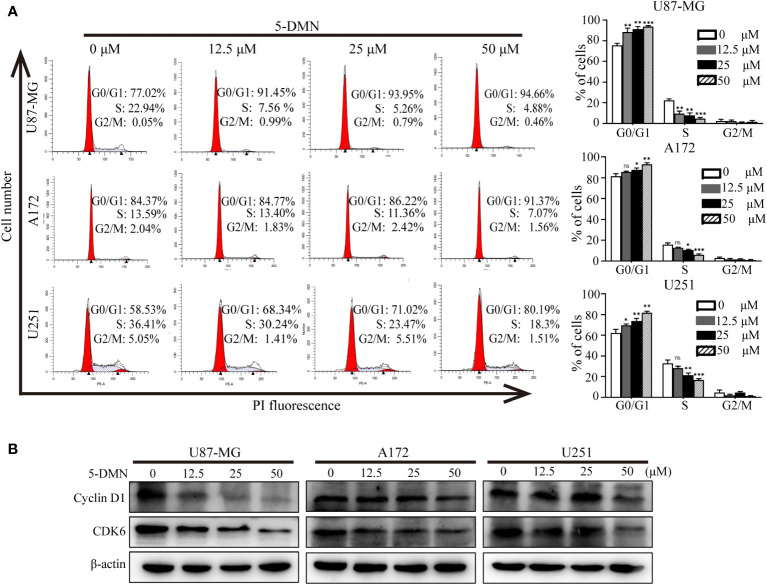
5-DMN induces cell cycle arrest at the G0/G1 phase in GBM cells. **(A)** The cell cycle distribution of U87-MG, A172, and U251 cells treated with different concentrations of 5-DMN was determined using flow cytometry. The histograms indicated the distribution of cell cycle from three separate experiments. **(B)** Levels of Cyclin D1 and CDK6 proteins in 5-DMN-treated GBM cells were detected by western blotting. The loading control was β-actin. *p<0.05, **p<0.01, ***p<0.001. ns, no significance.

To investigate whether 5-DMN-induced decrease in GBM cell viability was related to cell cycle arrest, flow cytometry was used to analyze cell cycle distribution after a 48-h treatment with 5-DMN. **Figure 2A** showed that the percentages of 5-DMN treated cells in the G0/G1 phase was considerably higher than that the control group. To further explained the mechanism that 5-DMN mediated cell cycle arrest, the expression of G0/G1 phase related proteins was evaluated in U87-MG, A172 and U251 cells, respectively. The activity of Cyclin D1 and CDK are required for G1 phase progression of the cell cycle and G1/S transition (20). Western blotting analysis demonstrated that Cyclin D1 and CDK6 expression was considerably reduced in 5-DMN-treated GBM cells compared with the control group (**Figure 2B**). The aforementioned data showed that 5-DMN induced G0/G1 phase cell cycle arrest by regulating the expression of Cyclin D1 and CDK6.

### 3.3 5-DMN induces apoptosis in GBM cells

**Figure 3:**
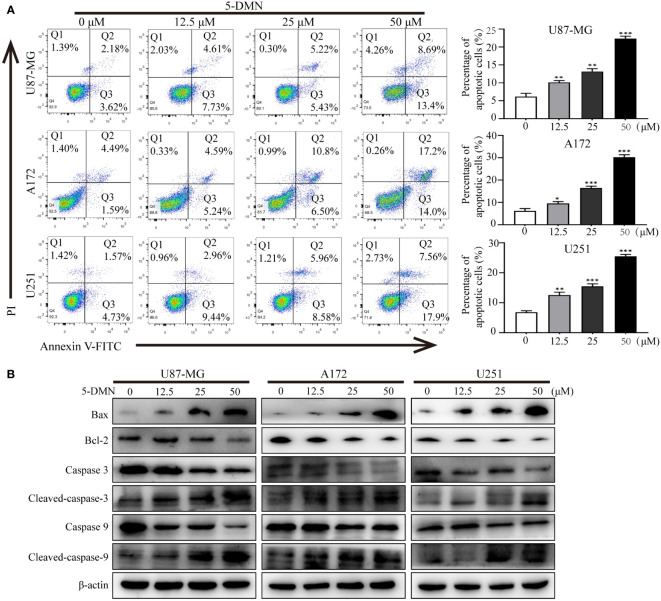
5-DMN induces GBM cells apoptosis. **(A)** The apoptotic rate of U87-MG, A172, and U251 cells after 5-DMN treatment was determined using double staining with Annexin V-FITC/PI. The histograms showed the percentage of apoptosis in three separate experiments. **(B)** Western blotting analysis of the expression of Bcl-2, Bax and caspase-3/9. The loading control was β-actin. *p<0.05, **p<0.01, ***p<0.001 vs. the untreated group cells.

To further investigate whether the inhibitory activity of 5-DMN-induced cell proliferation was associated with apoptosis, Annexin V-FITC/PI double staining was used to evaluate the apoptotic rate of cells treated with 5-DMN for 48 h. As shown in **Figure 3A**, the apoptotic cells were significantly increased in U87-MG, A172 and U251 cells with the increase of 5-DMN concentration. Then, the expression of signaling proteins related to apoptosis was tested by western blotting in these cells. We found that the protein levels of Bax, cleaved caspase-3, -9 was remarkedly elevated, while the expression of Bcl-2, caspase-3, -9 was inhibited in 5-DMN-treated cells (**Figure 3B**). In conclusion, these results demonstrated that 5-DMN effectively induced GBM cells apoptosis.

### 3.4 5-DMN inhibits the ERK1/2, AKT and STAT3 signaling pathways in GBM cells

**Figure 4:**
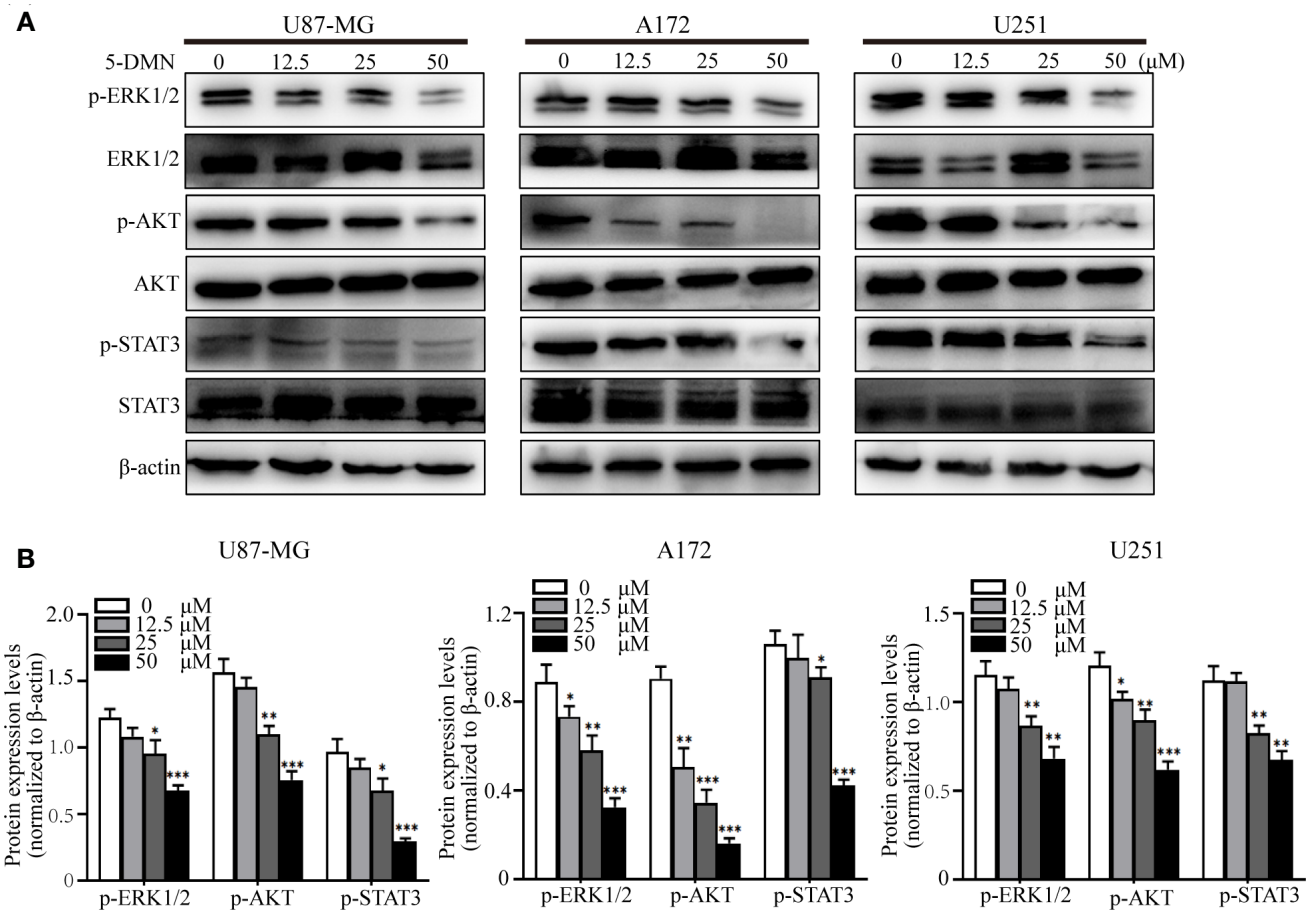
5-DMN inhibits the ERK1/2, AKT and STAT3 pathways in GBM cells. Cultured U87-MG, A172 and U251 cells were incubated with or without 50 µM 5-DMN for 48 h. **(A)** Western blotting analysis of the expression of p-ERK1/2, ERK1/2, p-AKT, AKT, p-STAT3 and STAT3. **(B)** The relative protein levels of p-ERK1/2, p-AKT and p-STAT3 in 5-DMN-treated GBM cells were analyzed. The protein levels in the untreated group were used to standardize the relative protein ratio. *p<0.05, **p<0.01, ***p<0.001 vs. cells in the untreated group.

ERK1/2, PI3K/AKT and STAT3 signaling are classical oncogenic signaling pathways that have been reported in GBM (21, 22). To tested whether 5-DMN had the inhibitory effect on these signal molecules, we treated GBM cells with 5-DMN at four different concentrations. Western blotting analysis showed that the phosphorylation of ERK1/2, AKT and STAT3 in 5-DMN treated cells were considerably lower than the control group (**Figures 4A, B**). These data indicated that 5-DMN could inhibit the ERK1/2, PI3K/AKT and STAT3 signal pathway in GBM cells.

### 3.5 Cell cycle arrest and apoptosis are mediated by the inhibition of the ERK1/2, AKT and STAT3 signaling pathway

**Figure 5:**
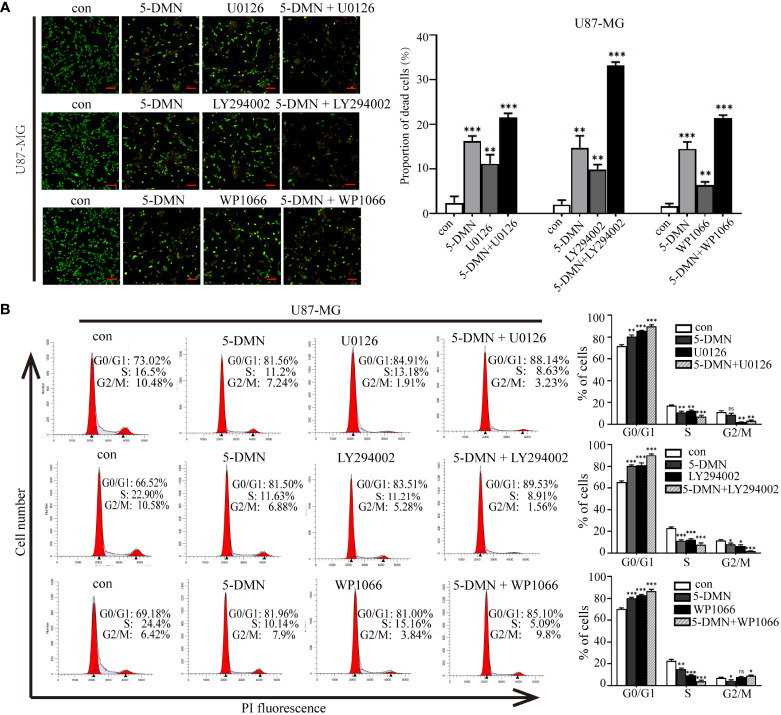
Roles of ERK1/2, AKT and STAT3 signaling in cell viability and G0/G1 phase arrest triggered by 5-DMN. **(A)** Live/dead staining assay of U87-MG cells after 5-DMN (50 μM) with or without the U0126 (15 μM), LY294002 (15 μM) and WP1066 (10 μM) for 48 h. **(B)** U87-MG cells were treated 5-DMN with or without the inhibitors and cell cycle distribution was determined. *p<0.05, **p<0.01, ***p<0.001 vs. cells in the control group. ns, no significance.

**Figure 6:**
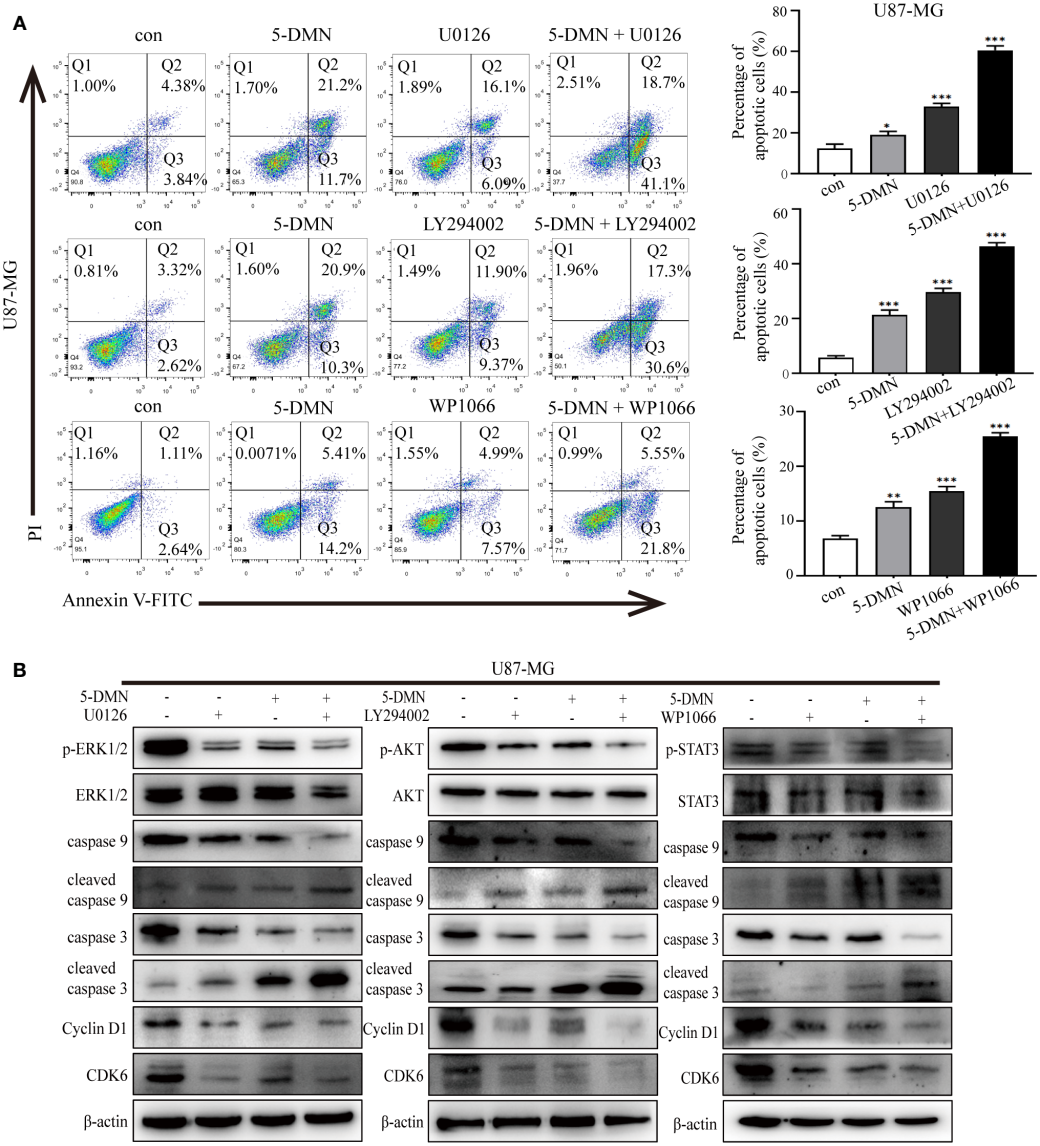
The ERK1/2, AKT and STAT3 signaling pathways influence the effects of 5-DMN on cell apoptosis, cycle and apoptosis-related proteins. **(A)** The apoptotic rate of U87-MG cells was determined using Annexin V-FITC/PI double staining after 5-DMN treatment with or without inhibitor. **(B)** Western blotting detected cell cycle proteins and apoptosis-related proteins levels in 5-DMN-treated U87-MG cells with or without pathway inhibitors. *p<0.05, **p<0.01, ***p<0.001 vs. cells in the control group.

To determine whether 5-DMN induced cell viability, cell cycle arrest and apoptosis were related to ERK1/2, AKT or STAT3 signaling pathways, the MEK inhibitor U0126, PI3K inhibitor LY294002 and STAT3 inhibitor WP1066 were used for further investigation in U87-MG cells. **Figure 5A** showed that the number of PI-positive cells in the U0126, LY294002 and WP1066 groups were obviously higher than the control group. Interestingly, a higher number of PI-positive cells were observed when combination of these inhibitors and 5-DMN than for 5-DMN treatment. Flow cytometric analysis also demonstrated that U0126, LY294002 and WP1066 significantly increased G0/G1 phase arrest induced by 5-DMN (**Figure 5B**). The same results were obtained by using flow cytometry to detect the apoptotic rate in 5-DMN or combination different inhibitors treated U87-MG cells (**Figure 6A**). These results indicated that in the process of G0/G1 phase arrest and cell death, ERK1/2, AKT and STAT3 signaling were suppressed by 5-DMN. Western blotting revealed that U0126, LY294002 and WP1066 further enhanced the inhibition of 5-DMN on the expression of cyclin-related proteins (Cyclin D1 and CDK6) and the promotion on the expression of pro-apoptosis-related proteins (caspase-3 and -9) (**Figure 6B**). In summary, 5-DMN induced G0/G1 phase arrest and triggered apoptosis by blocking the ERK1/2, AKT and STAT3 signaling pathways in GBM cells.

### 3.6 5-DMN inhibits the proliferation of GBM cells *in vivo*

**Figure 7:**
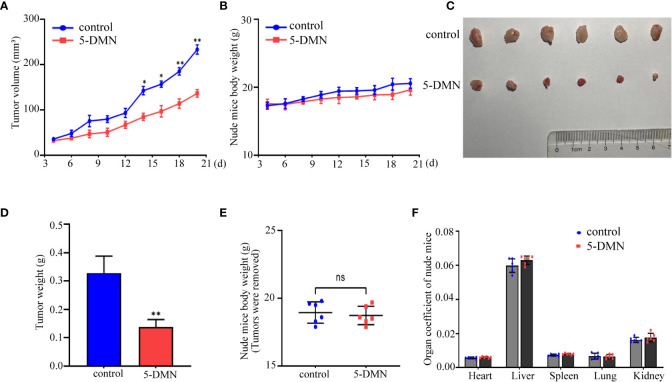
The antitumor effect of 5-DMN in BALB/c nude mice tumor model. **(A)** The volumes of tumor were evaluated at the indicated number of days after animals were treatment with 5-DMN. **(B)** The body weight changes of 5-DMN-treated group or control group mice. **(C)** Photograph of tumor tissues isolated from killed nude mice. **(D)** Quantification of tumor weight. Body weights **(E)** and organs weights **(F)** of nude mice were measured after tumors were resection. *p<0.05, **p<0.01 vs. cells in the untreated group. n= 6. ns, no significance.

To further assess the cytotoxicity of 5-DMN *in vivo*, U87-MG cells (5 × 10^6^ cells/100 µL) were implanted into BALB/c nude mice *via* slowly injection. When the tumor grew to a diameter of 3-4 mm, nude mice were randomly assigned to the control and 5-DMN groups, and then injected intraperitoneally every other day. After nine times of administration, the tumor growth curve results showed that 5-DMN (3 mg/kg) effectively inhibited the tumor growth compared with the control group (**Figure 7A**). During treatment, the weight of nude mice was monitored before each injection, and the results demonstrated that 5-DMN treatment had no influence on body weight (**Figure 7B**). Tumors were separated and tumor weights were also measured (**Figures 7C, D**). The tumor weight of the animals in 5-DMN group was 0.135 ± 0.027 g, significantly less than that of 0.317 ± 0.043 g in the control group. The aforementioned results suggested 5-DMN treatment group could delay tumor growth compared with the control group. During the tumorigenesis period, there was no mice death in the treatment group or the control group. To evaluate the drug toxicity of 5-DMN on mice at the therapeutic dose, the weights of body and organs weights were also measured when tumors were removed. As shown in **Figures 7E, F**, the results demonstrated there were no statistical different between 5-DMN and control groups. Therefore, the results showed that 5-DMN, which might have limited toxicity *in vivo*, could significantly inhibited the growth of GBM xenograft.

## 4 Discussion

Glioblastoma (GBM) is the most common primary malignant brain tumor with the characteristics of high recurrence rate, high mortality and the median survival of less than one year (23). Although 5-DMN has been implicated in the inhibition of cell proliferation in some cancers (12–14, 24), the underlying molecular mechanism which 5-DMN inhibits glioblastoma progression remains unclear. According to our findings, 5-DMN decreased the growth of GBM cell lines both *in vitro* and *in vivo*. This inhibition of 5-DMN was associated with cell cycle arrest and enhanced apoptosis. Furthermore, we also observed that 5-DMN has the capacity to prevent GBM cell migration and invasion. The underlying mechanisms of 5-DMN action in GBM cells may be influenced by the inhibitory effect of 5-DMN on the ERK1/2, PI3K/AKT and STAT3 signaling pathway.

Recent research have indicated that 5-DMN induced cytotoxicity in a variety types of cancer cells; however, the pharmacokinetics of 5-DMN differ in different tissues, which may lead to different antitumor effects (18). In *in vitro* studies, 5-DMN significantly decreased cancer cell line viability, including HCT116, THP1, CL1-5 and CL13, which IC50 values were 13.5 μM, 32.3 μM, 12.8 μM and 21.8 μM, respectively (6, 15, 16). Our results showed that 5-DMN exhibited different cytotoxicity in U87-MG, A172 and U251 cells. A172 and U251 cells treated with 5-DMN for 48 h had a similar IC50 values (50 μM) whereas the IC50 values in U87-MG cells were 33.5 μM. In addition, the high death rates and low cure rates of cancer are due in large part to the highly aggressive nature of tumor cells (25). Our study showed that 5-DMN could inhibit the migratory and invasive potential of cultured three different GBM cells.

The G1/S phase checkpoint is the first important restriction point in the cell division cycle (26). Cyclin-dependent kinases (CDKs) belongs to the serine/threonine protein kinase family, which regulate cell division and transcription (27). Unrestricted cell cycle progression and rapid growth caused by abnormal CDK4/6 signaling have been confirmed as glioma progression markers (28). Cyclin D1, a D-type cyclin protein, is an important regulator of cell cycle progression. Recent research found that Cyclin D1 expression was significantly higher in GBM tissues, predicting poor outcomes (29). Cyclin D1/CDK4 (CDK6) form complexes and then responsible for early G1 regulation (30). 5-DMN was discovered to block cell cycles in both the G0/G1 and G2/M phases of HCT116 (p53^+/+^), while in THP-1 and lung cancer cells, 5-DMN induced S- and G2/M phase arrest respectively (6, 8, 15, 31). Our results demonstrated that 5-DMN enhanced the percentage of G0/G1 phase in three GBM cells. A further western blotting analysis showed that 5-DMN was able to significantly downregulate the protein level of Cyclin D1 and CDK6 in concentration-dependent manner in GBM cells. Therefore, 5-DMN is a natural Cyclin D1 and CDKs inhibitor that suppress GBM tumorigenesis by inducting G0/G1 phase arrest. In addition, p21 and p27 are endogenous inhibitors of Cyclin/CDK and prevent the G1 transition to S by binding to the complex (32, 33). Whether p21 and p27 participate in the inhibition of 5-DMN-induced glioma cell proliferation remains to be further tested.

Apoptosis is programmed cell death that plays a critical role in the pathogenesis of GBM. Mitochondria-mediated caspase cascade activation is intimately associated with apoptosis, can be initiated by many stimuli (34). Members of the Bcl-2 family are generally considered to be located in the mitochondrial outer model and act as important regulators to regulate apoptosis (35). The Bcl-2 family is separated into two groups: One group contains anti-apoptotic proteins such as Bcl-2, while another group contains pro-apoptotic proteins such as Bax. The balance between these two proteins influences whether or not tumor cells undergo apoptosis (36, 37). Increasing evidence revealed that 5-DMN is a powerful apoptosis inducer in AML cell and non-small-cell lung carcinoma cells (6, 15). Consistent with previous research, GBM cell apoptosis related proteins were also changed after 5-DMN treatment, including caspase-9 and caspase-3 activation; the Bcl-2 was down-regulated, while the Bax was up-regulated. Therefore, 5-DMN can induce apoptosis of GBM cells through mitochondrial signaling pathway.

Inflammation is a stress response that is closely associated with the development of cancer (38). Previous study reported that 5-DMN reduced the expression of inflammation-related cytokines IL-1β, IL-6 and TNF-α by inhibiting the expression of p-JAK2 and p-STAT3 (39). Another study demonstrated that 5-DMN could reduce CCl4-induced fibrotic liver and regulate inflammatory responses in liver tissue *via* blocking the MAPK pathway (40). As we know, the JAK2/STAT3 and MAPK pathways also are classic crucial oncogenic signaling pathway, but whether 5-DMN regulates these two pathways in tumors has not been reported. Our study found that 5-DMN could significantly reduce the levels of p-ERK1/2 and p-STAT3 in a concentration-dependent manner in GBM cells. Furthermore, the inhibition of the ERK1/2 and STAT3 pathway by application U0126 and WP1066 could increase cell apoptosis and the number of cells blocked in G0/G1 phase. PI3K/AKT signaling not only regulates cell proliferation, but it also plays a role in tumor cell apoptosis (41). The PI3K/AKT pathway is also abnormally activated in GBM, as evidenced by abnormally elevated phosphorylation levels (42, 43). Although previous research found that the PI3K/AKT pathway was not required for autophagy in response to 5-DMN (6), our data showed that 5-DMN reduced the expression of p-AKT. Surprisingly, the PI3K/AKT pathway was also involved in the regulation of cell cycle arrest and apoptosis produced by 5-DMN. In summary, the above evidence indicated that 5-DMN promoted cell cycle arrest and apoptosis *via* inhibition of the ERK1/2, PI3K/AKT, and STAT3 signaling pathways.

Chen and Tan et al. reported that 5-DMN inhibited the growth of CL1-5 cells using murine ectopic xenografts models of lung cancer (6, 44). Our *in vivo* data demonstrated that the i.p. injection of 5-DMN at doses of 3 mg/kg, could greatly decrease tumor development in the nude mice transplantation model of GBM. Moreover, the body and organ weight data demonstrated that the nude mice treated with 5-DMN grow normally, which may imply that 5-DMN has low toxicity and less impact side effects *in vivo*. This may serve as a reminder that 5-DMN is a potentially effective anti-GBM drug, and we will investigate its toxicity in future experiments.

However, there are some limitations in our study. Although treatment with 5-DMN significantly decreased cell malignant behavior and promoted cell apoptosis, this phenomenon was only validated in three human GBM cell lines and subcutaneous tumor models. Further study will be required to better characterize this function. In conclusion, this study demonstrated that 5-DMN could inhibit cell proliferation, induce apoptosis in GBM cells *in vitro* and *in vivo* and effectively arrest the cell cycle in G0/G1 phase, probably by deactivating the ERK1/2, PI3K/AKT and STAT3 signaling pathways. According to the findings of this study, 5-DMN might be utilized as a potential drug for GBM treatment.

## Data availability statement

The original contributions presented in the study are included in the article/**Supplementary Material**. Further inquiries can be directed to the corresponding authors.

## Ethics statement

The animal study was reviewed and approved by the Animal Experiment Ethics Committee of Binzhou Medical University.

## Author contributions

XZhang analyzed and interpreted the experiments and wrote the manuscript. LZ and JX designed and analyzed the experiments. YW, YL and CZ performed cell cycle, western blotting analyses and the subcutaneous model *in vivo* assays. HZ and YZ performed the cell culture and cell behavior experiments. XZhu and YD assisted with data analysis and manuscript preparation. YD revised and approved the final version of the manuscript. All authors contributed to the article and approved the submitted version.

## Supplementary material

Supplementary Figure 1 The inhibitory effect of 5-DMN on normal cells viability and TMZ on GBM cells. **(A)** Cell viability of normal human brain astrocyte HA1800 and HEK293T cells after being treated with different concentrations of 5-DMN for 48 h. **(B)** Cell viability was determined using the MTT assay after cultured U87-MG, A172, and U251 cells were incubated with various doses (0, 6.25, 12.5, 25, 50, 100 μM) of TMZ for 48 h. **(C)** Wound healing assays were used to determine the migration ability of GBM cells after 48 h of incubation with TMZ (0, 25, 50 μM). Values are the means ± SD of three independent experiments. *p<0.05, **p<0.01, ***p<0.001 vs. cells in the untreated control group.
