# Corrigendum: 5-Demethylnobiletin mediates cell cycle arrest and apoptosis via the ERK1/2/AKT/STAT3 signaling pathways in glioblastoma cells

**DOI:** 10.3389/fonc.2024.1494738

**Published:** 2024-09-30

**Authors:** Xuehua Zhang, Leilei Zhao, Jinlong Xiao, Yudi Wang, Yunmeng Li, Chaoqun Zhu, He Zhang, Yurui Zhang, Xiao Zhu, Yucui Dong

**Affiliations:** ^1^ Department of Immunology, Binzhou Medical University, Yantai, China; ^2^ School of Computer and Control Engineering, Yantai University, Yantai, China; ^3^ Department of Immunology, Qiqihar Medical University, Qiqihar, China

**Keywords:** glioblastoma, 5-Demethylnobiletin, cell cycle arrest, cell apoptosis, ERK1/2, AKT, STAT3

In the published article, there was an error in **Figure 1D** as published. We regret in **Figure 1D** the pictures of A172 cells (25 μM) and U251 cells (50 μM) partially overlapped, when we sorted out the raw data. The picture of U251 cells (50 μM) is wrong. In the correct figure below, the picture representing U251 (50 μM) group in **Figure 1D** has been replaced. We used the data from the original images for statistical analysis. The corrected **Figure 1** and its caption appear below.

**Figure 1:**
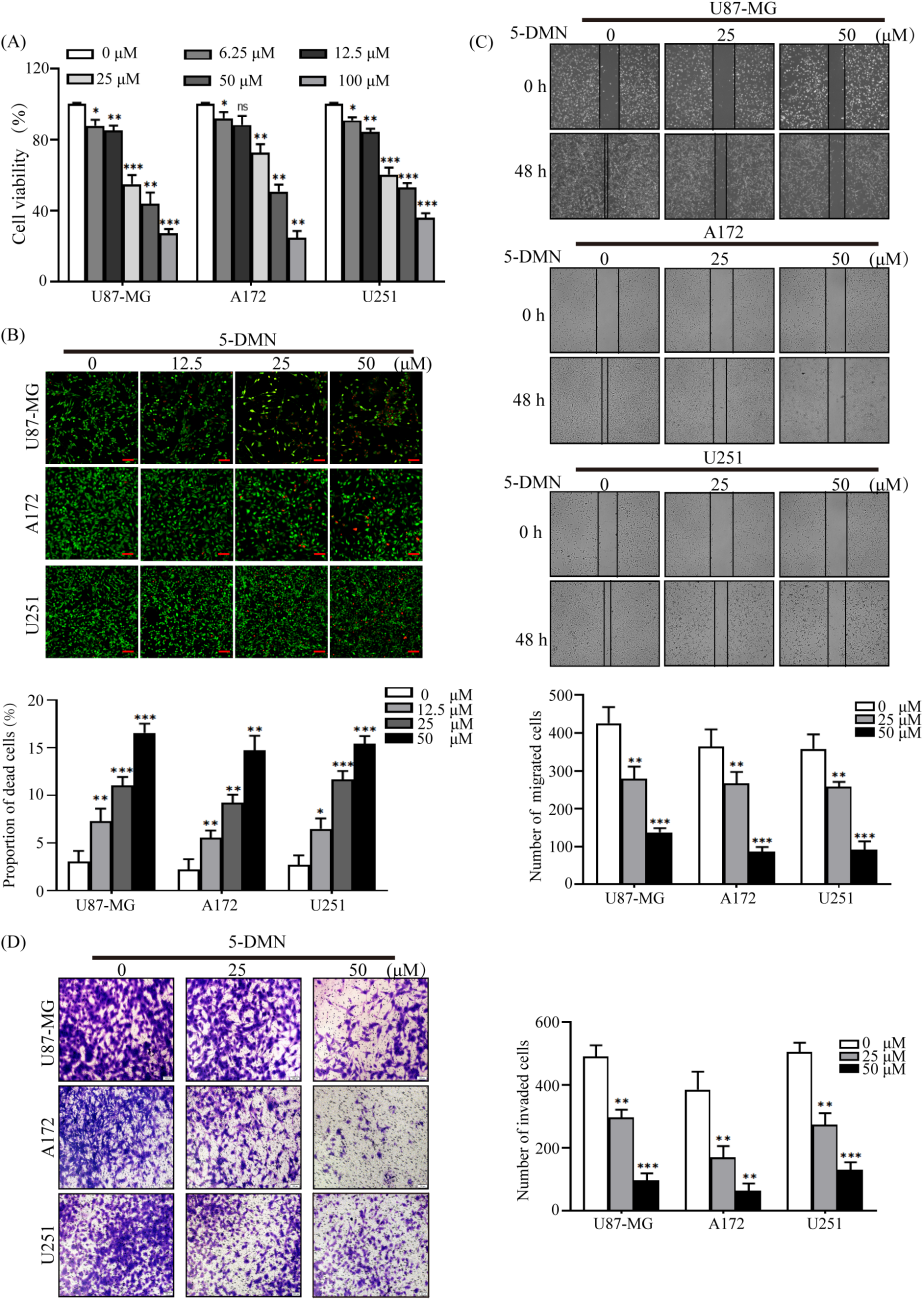
Inhibitory effects of 5-DMN on the viability, migration and invasion of GBM cells. **(A)** Cell viability was determined using the MTT assay after cultured U87-MG, A172, and U251 cells were incubated with various doses of 5-DMN for 48 h. **(B)** The merged image of live cells (green) and dead cells (red) in GBM cells treated with different concentrations of 5-DMN. Scale bar = 100 μm. **(C)** Wound healing assays were used to determine the migration ability of U87-MG, A172, and U251 cells after 48 h of incubation with 5-DMN (0, 25, 50 μM). **(D)** The invasion ability of GBM cells treated with or without 5-DMN (25, 50 μM) for 48 h was detected by transwell assays. Values are the means ± SD of three independent experiments. *p<0.05, **p<0.01, ***p<0.001 vs. cells in the untreated control group. ns, no significance.

The authors apologize for this error and state that this does not change the scientific conclusions of the article in any way. The original article has been updated.

