# Supplementary figures and images for: 5-Demethylnobiletin mediates cell cycle arrest and apoptosis via the ERK1/2/AKT/STAT3 signaling pathways in glioblastoma cells
Source: Front Oncol. 2023 Apr 17;13:1143664. doi: 10.3389/fonc.2023.1143664 (PMC10149914; doi:10.3389/fonc.2023.1143664)

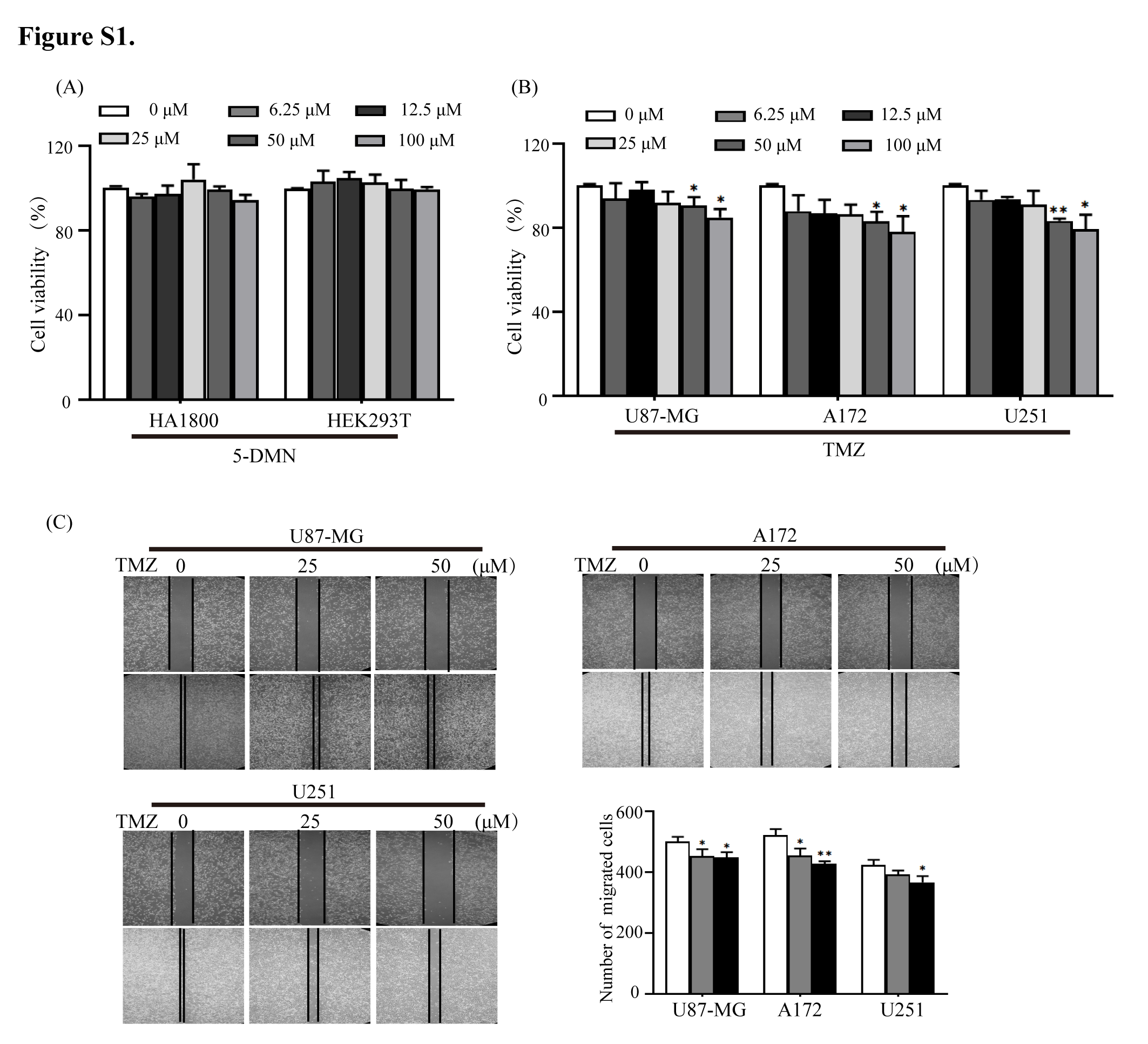

Supplement: Supplementary Figure 1 — The inhibitory effect of 5-DMN on normal cells viability and TMZ on GBM cells. (A) Cell viability of normal human brain astrocyte HA1800 and HEK293T cells after being treated with different concentrations of 5-DMN for 48 h. (B) Cell viability was determined using the MTT assay after cultured U87-MG, A172, and U251 cells were incubated with various doses (0, 6.25, 12.5, 25, 50, 100 μM) of TMZ for 48 h. (C) Wound healing assays were used to determine the migration ability of GBM cells after 48 h of incubation with TMZ (0, 25, 50 μM). Values are the means ± SD of three independent experiments. *p<0.05, **p<0.01, ***p<0.001 vs. cells in the untreated control group. [file Image_1.tif]
